# Effectiveness and safety of fluocinolone acetonide intravitreal implant in diabetic macular edema patients considered insufficiently responsive to available therapies (REACT): a prospective, non-randomized, and multicenter study

**DOI:** 10.1007/s10792-023-02864-2

**Published:** 2023-09-12

**Authors:** José María Ruiz-Moreno, Alfredo Adán, María Lafuente, Mónica Asencio Durán, Luís Arias Barquet, Alfredo García Layana, Javier Araiz Iribarren

**Affiliations:** 1 Puerta de Hierro-Majadahonda University Hospital, Joaquín Rodrigo, 2, 28222 Majadahonda, Madrid Spain; 2 Department of Ophthalmology, Castilla La Mancha University, Albacete, Spain; 3 Miranza, Spain; 4 Clínic University Hospital, Barcelona, Spain; 5 Virgen de la Arraixaca University Hospital, Murcia, Spain; 6 La Paz University Hospital, Madrid, Spain; 7 Bellvitge University Hospital, Barcelona, Spain; 8 Navarra University Clinic, Navarra, Spain; 9 Clinical Surgical Institute of Ophthalmology, Vizcaya, Spain

**Keywords:** Diabetic macular edema, Fluocinolone acetonide intravitreal implant, Corticoids, VEGF inhibitors

## Abstract

**Objective:**

To assess the effectiveness and safety of the intravitreal fluocinolone-acetonide implant (FAc-i) in patients with chronic diabetic macular edema who did not sufficiently respond to other available therapies.

**Methods:**

This was a multicenter, prospective, non-randomized, and phase-IV observational study conducted on patients with recurrent-DME who were insufficient responders to currently available therapies (REACT-Study). The primary end-point was the mean change in best-corrected-visual-acuity from baseline to month-24 values.

**Results:**

Thirty-one eyes from 31 patients were included in the study. Mean age was 68.0 ± 7.7 years, and 10 (32.3%) were women. Study patients had received 5.3 ± 7.3 previous DME treatments before starting the study. In the overall study sample, BCVA improved from 56.1 ± 12.3 letters at baseline to 62.4 ± 17.0 letters at month-24 (*p* = 0.0510). The eyes with a baseline BCVA < 70 ETDRS letters had a significant improvement in BCVA from 53.2 ± 10.2 letters at baseline to 61.5 ± 17.9 letters at month-24 (*p* = 0.0165). In the overall study population, central-subfoveal-thickness (CST) was significantly reduced from 474.0 ± 135.1 µm at baseline to 333.4 ± 135.6 at month-24 (*p* < 0.0001). Similarly, macular-volume (MV) was significantly reduced from 10.7 ± 2.7 mm^3^ at baseline to 9.6 ± 2.9 mm^3^ (*p* = 0.0027) at month-24. Among the 31 study eyes, 19 (61.3%) required an additional treatment for DME. Throughout the study, 9 (29.0%) eyes required ocular hypotensive medication for controlling their intraocular-pressure and 5 (16.1%) eyes underwent cataract surgery.

**Conclusions:**

In DME eyes who did not sufficiently respond to previous therapies, the FAc-i was associated with an improvement in visual and anatomic outcomes. There were no unexpected adverse-events.

**Trial registration number:**

EudraCT identifier: 2016-001680-37.

**Supplementary Information:**

The online version contains supplementary material available at 10.1007/s10792-023-02864-2.

## Introduction

Diabetic macular edema (DME) is a prevalent and disabling disease that directly impairs central vision of patients with diabetic retinopathy (DR), making diabetes the leading cause of severe vision impairment in working-age populations of developed countries [1].

Among the different treatment options currently available for patients with DME, vascular endothelial growth factor inhibitors (anti-VEGF) are considered first-line treatments [2]. However, in those eyes who do not adequately respond to anti-VEGF therapy or in those patients with systemic contraindications to anti-VEGF, intravitreal corticosteroid implants may be considered the treatment of choice [2–4].

Fluocinolone acetonide sustained-release intravitreal implant (FAc-i) (ILUVIEN^®^; Alimera Sciences, Hampshire, UK) is a non-erodible implant, which can deliver a low daily dose (0.2 µg per day) sustainedly of fluocinolone acetonide over a 3-year period [5]. The FAc-i was approved in different European countries for treating eyes with recurrent and recalcitrant DME, i.e., DME eyes which do not adequately respond to other therapies [6]. Additionally, in Europe, it is also indicated for preventing the onset of relapse episodes in eyes with recurrent non-infectious uveitis [7, 8].

The effectiveness and safety of FAc-i was first demonstrated in the pivotal phase-III randomized controlled FAME trials, which were conducted on patients with chronic DME who received, at least, one session of laser treatment [9, 10]. The results of these studies found that FAc-i provided a significant and sustained improvement in best corrected visual acuity (BCVA) [9, 10].

Diverse multicenter, non-randomized, and phase 4 studies were designed to assess the effectiveness and safety of FAc-i n daily clinical practice conditions [11–14]. The ILUVIEN Registry Safety Study (IRISS; NCT01998412) reported a significant improvement in BCVA, which was greater in eyes with a shorter duration of DME, with no unexpected adverse events appearing [13]. Similarly, the PALADIN study found significant improvements in both visual and anatomic outcomes, with a favorable safety profile [14]. Finally, the Retro-IDEAL study demonstrated a significant improvement of BCVA and reduction in central retinal thickness over a period of 3 years [11]. In summary, these studies have evidenced the favorable, long-term benefit-to-risk profile of the FAc-i in eyes with DME who did not achieve an adequate response to previous therapies [11–14].

The evidence evaluating the effectiveness and safety of the FAc-i in the Spanish clinical setting is scarce and reflects the experience of retrospective and single center studies [15, 16].

This study aimed to assess the effectiveness and safety of the FAc-i in patients with chronic DME who did not sufficiently respond to other available therapies.

## Methods

### Study design

Phase IV observational, prospective, non-randomized, multicenter, and national clinical trial conducted on eyes with chronic DME who were considered insufficiently responsive to available therapies (REACT).

This study was in accordance with the ICH guidelines and guidelines for Good Clinical Practice (GCP), with the Declaration of Helsinki (revised version, Fortaleza, October 2013) [17] and the local laws and guidelines of the countries in which the study is being conducted.

The study protocol was approved by the ethics committee of Bellvitge University Hospital and registered in the European Union Clinical Trials Register (EudraCT identifier: 2016-001680-37).

The study began on September 26, 2017, and ended on February 16, 2022.

### Study participants

Patients ≥ 18 years of age; diagnosed with DME according to investigator’s clinical evaluation and demonstrated using fundoscopic photography and spectral domain optical coherence tomography (SD-OCT); who were considered as insufficiently responsive to currently available therapies; and willing to comply with the investigators and protocol indications were included in the study.

The main inclusion/exclusion criteria are listed in Table S1.

### Study treatment

All eligible patients with a signed informed consent received a 190 µg FAc-i (ILUVIEN^®^; Alimera Sciences, Hampshire, UK) in applicator with an initial release rate of 0.2 µg per day. The implant was administered by injection according to the method of administration defined in the Summary of Product Characteristic [18]. Topical antibiotic was prescribed for all patients for 3–5 days following the day of implantation.

### Patient visits

This protocol includes one screening visit (− 14 to 0 days before FAc-i administration); one baseline visit (administration of FAc-i) and 10 follow-up visits performed at week 1 ± 2 days; month 1 ± 7 days; months-3, -6, -9, and − 12 ± 15 days; and months-15, - 18; -21; and − 24 ± 30 days.

### Outcomes

The primary end-point was the mean change in BCVA from baseline values.

The secondary end-points included the mean changes in central subfield thickness (CST) and macular volume (MV) assessed using SD-OCT; the incidence of adverse events; and the results of a Quality-of-Life Analysis (VFQ-25 questionnaire).

### Statistical analysis

A standard statistical analysis was performed using SAS/STAT software, Version 9.4 of the SAS System for Windows. Copyright^©^ 2023 SAS Institute Inc.

Prior to the study, it was planning to include 40 patients, 5 patients in each clinical center. Because it was a pilot study, no sample size was estimated.

Continuous variables were described by mean; median; standard deviation (SD); inter-quartile range (IqR); minimum; and maximum, as appropriate, while categorical variables were summarized by number and percentages.

No data were excluded due to protocol violations.

Intent-to-treat analysis included all patients who received study medication.

The two-tailed paired sample *t* test or the Wilcoxon test, as appropriate, were used to assess changes in BCVA, CST, MV, and intraocular pressure (IOP).

Categorical variables were compared using a Chi-square test and a Fisher`s exact test, as needed. *p* value of less than 0.05 was considered significant.

In order to evaluate the influence of the initial BCVA on the treatment effectiveness, the eyes were stratified according to their baseline BCVA (BCVA < 50 ETDRS letters versus BCVA ≥ ETDRS 50 letters).

## Results

Thirty-one eyes from 31 patients were included in the study. Eleven patients were lost of follow-up 10 for personal reasons (due to the lockdown measures taken during the SARS-COV-2 pandemic) and one patient due to an adverse event (Vitreous hemorrhage). The study flowchart is shown in Figure S1.

Mean age was 68.0 ± 7.7 years, and 10 (32.3%) were women. The median (IqR) follow-up was 35.9 (23.6 to 37.5) months.

Study patients had received 5.3 ± 7.3 previous DME treatments before starting the study.

The main demographic and clinical characteristics are summarized in Table 1.

**Table 1:** Main demographic and clinical characteristics of the study population

Variable	Overall (n = 31)
*Age, years*	
Mean ± SD	68.0 ± 7.7
Median (IqR)	68.5 (63.1 to 73.0)
*Sex, n (%)*	
Women	10 (32.3)
Men	21 (67.7)
*Study eye, n (%)*	
Right	16 (51.6)
Left	15 (48.4)
*Race, n (%)*	
Caucasian	31 (100.0)
*Lens status, n (%)*	
Phakic	10 (32.3)
Pseudophakic	21 (67.7)
*Type of DM, n (%)*	
Type 1	1 (3.2)
Type 2	30 (96.8)
*Length of DM, years*	
Mean ± SD	14.6 ± 10.2
Median (IqR)	17.0 (4.0 to 23.0)
*Length of DME, years*	
Mean ± SD	3.9 ± 3.2
Median (IqR)	3.0 (2.0 to 6.0)
*HbA1c, %*	
Mean ± SD	6.8 ± 0.9
Median (IqR)	6.7 (6.3 to 7.1)
Ophthalmic disease*	
DME	31 (100.0%)
DR	19 (61.3)
Previous cataract surgery	16 (54.6)
Previous PRP	15 (48.4)
Focal laser	5 (16.1)
Vitrectomized	3 (9.7)
Medical history, n (%)*	
None	8 (25.8)
Systemic hypertension	14 (45.2)
Musculoskeletal disease	8 (25.8)
CVD	7 (22.6)
Cancer	5 (16.1)
BPH	3 (9.7)
CKD	3 (9.7)
Respiratory disease	3 (9.7)
*Previous DME treatments*	
Mean ± SD	5.3 ± 7.3
Median (IqR)	3.0 (1.5 to 6.6)
*Previous DME treatments, n (%)*	
Previous laser	19 (61.3)
PRP	15 (48.4)
Focal	5 (16.1)
Anti-VEGF	26 (83.9)
Bevacizumab	10 (32.3)
Ranibizumab	14 (45.2)
Aflibercept	12 (38.7)
Corticoids	20 (64.5)
Triamcinolone	1 (3.2)
Dexamethasone	19 (61.3)
Both	1 (3.2)
BCVA**	
Mean ± SD	56.1 ± 12.3
Median (IqR)	56.0 (49.0 to 66.0)
CST, µm	
Mean ± SD	474.0 ± 135.1
Median (IqR)	458.0 (366.0 to 579.0)
*MV, mm*^*3*^	
Mean ± SD	10.7 ± 2.7
Median (IqR)	10.9 (9.3 to 12.1)
*VFQ-25*	
Mean ± SD	69.7 ± 20.3
Median (IqR)	76.5 (48.3 to 86.6)
*IOP, mmHg*	
Mean ± SD	14.6 ± 3.0
Median (IqR)	15.0 (12.0 to 16.0)

^*^The total sum may be greater than 100%, since patients may have more than one systemic pathology

^**^Early Treatment Diabetic Retinopathy Study (ETDRS) letters

*SD* Standard deviation; *IqR* Interquartile range; *DM* Diabetes Mellitus; *DME* Diabetic macular edema; *DR* Diabetic retinopathy; *PRP* Panretinal photocoagulation; *CVD* Cardiovascular disease; *BPH* Benign prostatic hyperplasia; *CKD* Chronic kidney disease; *anti-VEGF* Vascular endothelial growth factor inhibitors; *BCVA* Best corrected visual acuity; *CST* Central subfoveal thickness; *MV* Macular volume; *VFQ* Visual Function Quality

### Effectiveness analysis

#### Best corrected visual acuity

**Fig. 1:**
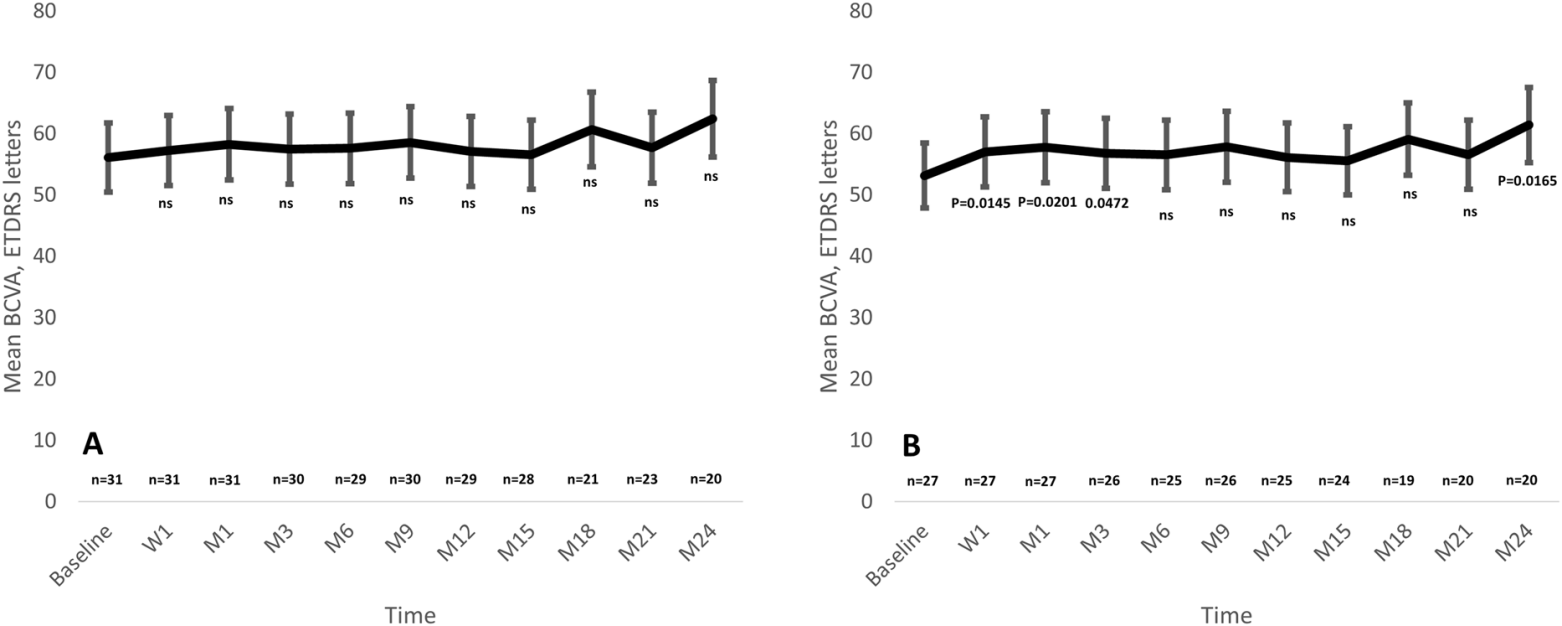
Mean best corrected visual acuity throughout study follow-up in the overall study population (**A**) and in the eyes with a baseline BCVA < 70 ETDRS letters (**B**). Statistically significance was calculated by using paired sample two-tailed t test or Wilcoxon test, as appropriate. *BCVA* Best corrected visual acuity; *ETDRS* Early Treatment Diabetic Retinopathy Study; *ns* Not significant; *W* Week; *M* Month

In the overall study sample, BCVA improved from 56.1 ± 12.3 letters at baseline to 62.4 ± 17.0 letters at month-24, although such improvement was not statistically significant (*p* = 0.0510) (Fig. 1A). Although baseline BCVA increased after FAc-i treatment at all the different time-point measured, such increase was not statistically significant (Figure S2A).

Nevertheless, in those eyes with a baseline BCVA ≤ 70 ETDRS letters, there was a significant improvement from 53.2 ± 10.2 letters at baseline to 61.5 ± 17.9 letters at month-24 (*p* = 0.0165) (Fig. 1B). As compared to baseline values, mean BCVA improvement was statistically significant at week-1, month-1, month-3, and month-24 in the eyes with a baseline BCVA 70 ETDRS letters (Figure S2B).

**Fig. 2:**
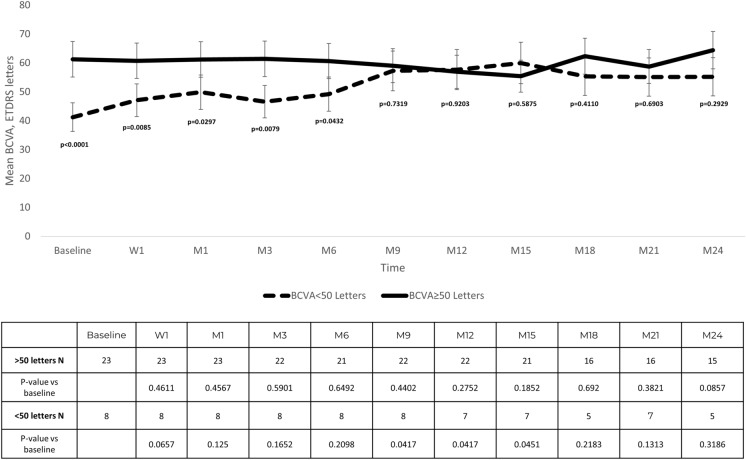
A comparison of the mean best corrected visual acuity (BCVA) throughout study follow-up in the eyes with a baseline BCVA < 50 ETDRS letters (8 eyes; dotted line) and those with a baseline BCVA ≥ 50 ETDRS letters (23 eyes; solid line).Statistically significance was calculated by using independent sample t test. *BCVA* Best corrected visual acuity; *ETDRS* Early Treatment Diabetic Retinopathy Study; *ns* Not significant; *W* Week; *M* Month

At baseline, 23 (74.2%) eyes had a BCVA ≥ 50 ETDRS letters and 8 (25.8%) ones had a BCVA < 50 ETDRS letters. Besides baseline BCVA, which was significantly lower in the eyes with BCVA < 50 ETDRS letters (*p* < 0.0001); BCVA was significantly greater at week-1 (*p* = 0.0085), month-1 (*p* = 0.0297), month-3 (*p* = 0.0079), and month-6 (*p* = 0.0432) in the eyes with a baseline BCVA ≥ 50 ETDRS letters (Fig. 2).

As compared to baseline values, BCVA improvement was statistically significant at months 9, 12, and 15 in the eyes with baseline BCVA < 50 ETDRS letters. Whereas, in those eyes with a baseline BCVA ≥ 50 ETDRS letters, BCVA remained stable throughout the study follow-up (Table S2).

#### Anatomic outcomes

**Fig. 3:**
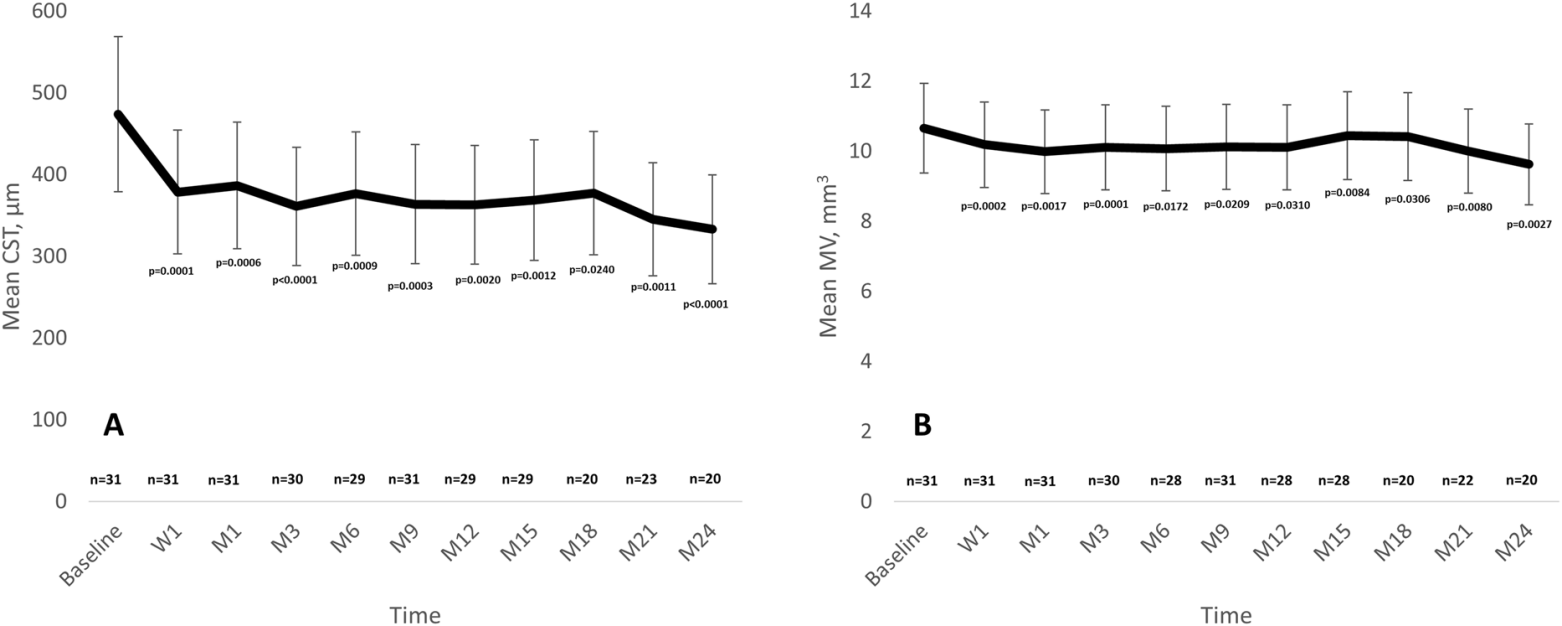
Mean central subfoveal thickness CST (**A**) and macular volume (MV) (**B**) throughout study follow-up in the overall study population. Statistically significance was calculated by using the two-tailed paired sample t test. *CST* Central subfoveal thickness; *MV* Macular volume; *W* Week; *M* Month

In the overall study population, CST was significantly reduced from 474.0 ± 135.1 µm at baseline to 388.7 ± 136.6 µm (*p* = 0.0001); 386.8 ± 132.4 µm (*p* = 0.0006); 361.4 ± 125.0 µm (*p* < 0.0001); 376.9 ± 154.4 µm (*p* = 0.0009); 364.0 ± 126.4 µm (*p* = 0.0003); 363.2 ± 162.6 µm (*p* = 0.0020); 368.7 ± 154.6 µm (*p* = 0.0012); 377.5 ± 146.6 µm (*p* = 0.0240); 345.6 ± 158.8 µm (*p* = 0.0011); and 333.4 ± 135.6 µm *p* < 0.0001) at week-1; and months 1, 3, 6, 9, 12, 15, 18, 21, and 24, respectively (Fig. 3A).

An analysis of the anatomical response based on the baseline BCVA revealed that, in the eye with a baseline BCVA ≥ 50 letters, CST was significantly reduced from 464.30 ± 133.4 µm at baseline to 321.05 ± 121.7 µm at month-24 (*p* = 0.0014) (Figure S3).

Although in eyes with a baseline BCVA < 50 letters the CST was reduced from 501.75 ± 145.3 µm at baseline to 375.40 ± 185.6 µm at month-24, such a difference was not statistically significant (*p* = 0.0625) (Figure S3).

Regarding MV, there was a statistically significant reduction in MV from 10.7 ± 2.7 mm^3^ at baseline to 10.2 ± 2.6 mm^3^ (*p* = 0.0002); 10.0 ± 2.7 mm^3^ (*p* = 0.0017); 10.1 ± 2.0 mm^3^ (*p* = 0.0001); 10.1 ± 1.9 mm^3^ (*p* = 0.0172); 10.1 ± 1.8 mm^3^ (*p* = 0.0209); 10.1 ± 2.4 mm^3^ (*p* = 0.0310); 10.5 ± 2.7 mm^3^ (*p* = 0.0084); 10.4 ± 2.4 mm^3^ (*p* = 0.0306); 10.0 ± 2.3 mm^3^ (*p* = 0.0080); and 9.6 ± 2.9 mm^3^ (*p* = 0.0027) at week 1; and months 1, 3, 6, 9, 12, 15, 18, 21, and 24, respectively (Fig. 3B).

### Rescue medication

Among the 31 study eyes, 19 (61.3%) required an additional treatment for DME. Fifteen (78.9%) eyes underwent intravitreal anti-VEGF and 11 (57.9%) an intravitreal corticoid (Table S3).

### Safety

Over the course of the study, 33 adverse events (AEs) were reported in 17 patients. Among them, 8 (24.2%) AEs were possible-definitive related to the study medication.

Six (18.2%) AEs were classified as serious, namely one retinal detachment, one colorectal cancer, one patient who underwent intestinal surgery, and 3 eye who developed cataract in the treated eye. Fifteen (45.5%) AEs were considered as moderate-to-severe. Additionally, 6 (19.4%) patients underwent cataract surgery throughout the study, 5 (16.1%) of them in the study eye.

An overview of the different AEs is shown in Table 2.

**Table 2:** Overview of the different adverse events that occurred throughout the study

	Overall (33 AEs/17 patients)*	
Eye disorders (study eye) (15 AEs/10 patients)	Cataract	5/5 (16.1%)
	Visual acuity reduced	2/2 (6.5%)
	Dry eye	1/1 (3.2%)
	Eye allergy	1/1 (3.2%)
	Eye pain	1/1 (3.2%)
	Glaucoma	1/1 (3.2%)
	Keratitis	1/1 (3.2%)
	Retinal detachment	1/1 (3.2%)
	Posterior capsule opacification	1/1 (3.2%)
Eye disorders (no study eye) (5 AEs/3 patients)	Cataract	1/1 (3.2%)
	Vitreous hemorrhage	1/1 (3.2%)
	Dry eye	1/1 (3.2%)
	Eye allergy	1/1 (3.2%)
	Diabetic retinal edema	1/1 (3.2%)
Investigations (5 AEs/5 patients)	Intraocular pressure increased	3/3 (9.7%)
	Intraocular pressure test	1/1 (3.2%)
	Intraocular pressure decreased	1/1 (3.2%)
Infections and infestations (3 AEs/3 patients)	COVID-19	2/2 (6.5%)
	Rhinitis	1/1 (3.2%)
Injury, poisoning, and procedural complications (2 AEs/2 patients)	Rib fracture	1/1 (3.2%)
	Ankle fracture	1/1 (3.2%)
Surgical and medical procedures (2 AEs/2 patients)	Gastrointestinal surgery	1/1 (3.2%)
	Blepharoplasty	1/1 (3.2%)
Neoplasms benign, malignant, and unspecified (1 AEs/1 Patient)	Colorectal cancer	1/1 (3.2%)
General disorders and administration site conditions (1 AEs/1 Patient)	Edema	1/1 (3.2%)
Reproductive system and breast disorders (1 AEs/1 Patient)	BPH	1/1 (3.2%)

^*^Number of events/number of patients

*AEs* Adverse events; *COVID* Coronavirus disease; *BPH* Benign prostatic hyperplasia

There was statistically significant increase in mean IOP from 14.6 ± 3.0 mmHg at baseline to 16.8 ± 2.7 mmHg (mean difference: 2.2 ± 2.9 mmHg; 95%CI: 0.6 to 3.9 mmHg; *p* = 0.0086). Five (16.1%) eyes had an increase in IOP ≥ 10 mmHg.

Throughout the study, 9 (29.0%) eyes required ocular hypotensive medication for controlling their IOP (one eye was already taken IOP lowering medication at baseline). Regarding the ocular hypotensive medications administered during the study, 5 (16.1%) eyes received timolol maleate 0.5%; 4 (12.9%) eyes received dorzolamide/timolol fixed combination; 3 (9.7%) eyes received brimonidine/timolol fixed combination; 3 (9.7%) eyes received latanoprost 0.005%; 3 (9.7%) eyes received brimonidine; and 2 (6.5%) eyes receive apraclonidine; and 2 (6.5%) eyes received treatment with brinzolamide.

## Discussion

Although DME is a disabling disease, which significantly impacts on patients’ quality of life, there are currently diverse therapeutic strategies for its management [2].

The advent of anti-VEGF meant a change in the paradigm of DME treatment [2]. However, there is a proportion, which can reach 40% of cases, that does not respond adequately to this treatment [19]. Moreover, many patients received suboptimal anti-VEGF treatment in real-life conditions [20].

The increasingly relevance that inflammation plays in the pathophysiology of DME has led to corticosteroids gaining importance as an alternative therapeutic strategy, particularly in recurrent DME and in patients resistant to anti-VEGF treatment [21, 22].

Sustained-release corticoid formulations have emerged as a therapeutic strategy that allows a gradual corticoid release, with the subsequent reduction in the patient’s treatment burden [2].

There are two sustained-release corticosteroid implants currently approved in Spain for treating patients with DME, namely the dexamethasone intravitreal implant 0.7 mg (Ozurdex^®^, AbbVie) and the fluocinolone acetonide intravitreal implant 0.19 mg (ILUVIEN^®^; Alimera Sciences, Hampshire, UK).

The current study aimed to evaluate the effectiveness and safety of the FAc-i in eyes with chronic DME who had an insufficient respond to other available therapies.

As far as we know, the REACT study is the first prospective, non-randomized, multicenter, national, and observational phase IV clinical trial evaluating the visual, anatomic, and safety outcomes of the FAc-i in Spain.

According to the results of this study, mean BCVA improved after FAc-i treatment at all the different time-point measured, although such increase was not statistically significant in the overall study sample. This fact was mainly due to the lockdown measures taken during the SARS-COV-2 pandemic for reducing the risk of infection spreading, which disrupted dramatically the provision of health care resulting of deferral of routine ophthalmic procedures [23, 24].

Nevertheless, in those eyes with a baseline BCVA < 70 ETDRS letters, BCVA improvement at month 24 was statistically significant (Mean BCVA improvement: 6.2 letters; *p* = 0.0165).

Furthermore, after stratifying patients by baseline BCVA, a significant improvement in BCVA was observed at months 9, 12, and 15 in the eyes with baseline BCVA < 50 ETDRS letters. While in those eyes with a baseline BCVA ≥ 50 ETDRS letters, BCVA remained stable throughout the study follow-up.

Despite the anatomic improvement observed in both groups after FAc-i treatment, this improvement was greater in the eyes with a baseline BCVA ≥ 50 letters. It might be hypothesized that eyes with a worse baseline BCVA would present more severe structural anatomical abnormalities, which would justify the differences in BCVA throughout the study between both groups [25, 26]. These findings clearly suggest a positive benefit to risk profile in patients treated earlier with the FAc-i.

Regarding the anatomic outcomes, as compared to baseline values, both CST and MV were significantly reduced at all the different time-points measured in the overall study population.

The effectiveness, in terms of visual and anatomic outcomes, of the FAc-i in patients with DME has been previously reported in different studies [6, 9–15, 25–34]. According to the results of a systematic-review and meta-analysis, the FAc-i provided a mean peak visual improvement of + 8.7 letters (range: 0.4 to 18.8 letters, median + 8.0 letters) and a maximum CRT reduction of − 34.3% (range: − 10.7% to − 55.8%, median: − 36.2%) from baseline [35].

Regarding visual outcomes, there does not seem to be a big difference between the real-life studies [36] and the FAME studies [9, 10] and the current study.

In the current study, BCVA improved by 4.5 letters at month-24, while the FAME study reported a BCVA improvement of 4.4 letters at month-24 [9] and a meta-analysis of real-world studies observed a visual acuity improvement of 4.5 letters at month-24 [36].

Similarly, the anatomical results found in the REACT study are in line with the literature published to date [6, 9–15, 25–36].

Nineteen (61.3%) eyes required an additional treatment for DME during the study. This result was greater than that reported by the FAME study (15.2%) [9, 10] and the meta-analysis (30% and 39%, respectively) [35, 36]. This finding might be related to the SARS-COV-2 pandemic, since the different measured adopted for controlling virus spreading led to deferral of routine ophthalmic procedures [23, 24].

Regarding safety, the REACT study has found no unexpected adverse events. Throughout the study, 33 adverse events (AEs) were reported in 17 patients, with 6 AEs considered as serious.

Over the course of the study, 8 (25.8%) eyes started ocular hypotensive medication for controlling their IOP, although none of them required IOP-lowering surgery.

Among the phakic eyes, 5 (50.0%) eyes underwent cataract surgery throughout the study.

These findings did not significantly differ from those published in the literature [6, 9–15, 25–36].

This study has some limitations that need to be considered when interpreting its results. The main one was the sample size. Although it was originally planned to include 40 eyes (5 eyes per center), only 31 eyes were included in the study. This could have motivated the lack of statistical significance in the BCVA improvement of the BCVA achieved at 24 months.

## Conclusions

FAc-i was associated with a trend toward improvement in BCVA at month 24 in the overall study sample. Moreover, in those eyes with a baseline BCVA < 70 ETDRS letters, there was a statistically significant improvement on BCVA at month 24 after FAc-i injection.

The anatomic outcomes, both the CST and the MV, improved significantly after the administration of the FAc-i.

Regarding safety, no unexpected adverse events were reported. Five (16.7%) eyes required cataract surgery, and 8 (25.8%) eyes started ocular hypotensive medication for controlling their IOP.

### Supplementary Information

Below is the link to the electronic supplementary material.

Study Flowchart. 31 patients were included in the study from September 26, 2017, to October 30, 2018, and all patients were treated. The patients participated in this study between 26 September 2017 (first consent) and 16 February 2022 (last follow-up visit). Eleven did not complete the study: 10 loss of follow-up and 1 due to an adverse event.

Mean change in best corrected visual acuity (BCVA) throughout study follow-up in the overall study population (A) and in the eyes with a baseline BCVA < 70 ETDRS letters. Statistically significance was calculated by using paired sample two-tailed t test or Wilcoxon test, as appropriate. BCVA: Best corrected visual acuity; ETDRS: Early Treatment Diabetic Retinopathy Study; ns: Not significant; W: Week; M: Month.

A comparison of the mean central subfoveal thickness CST throughout study follow-up in the eyes with a baseline BCVA < 50 ETDRS letters (8 eyes; gray columns) and those with a baseline BCVA ≥50 ETDRS letters (23 eyes; black columns). Intragroup statistically significance was calculated by using the Wilcoxon test. Between group differences were calculated with the independent-samples t test. CST: Central subfoveal thickness; MV: Macular volume; W: Week; M: Month; ns: Not significant. a Between group significance, †p<0.05 from baseline. ‡*p* < 0.01 from baseline. *p < 0.005 from baseline. **p < 0.001 from baseline.

Supplementary file4 (DOCX 15 kb)

Supplementary file5 (DOCX 17 kb)

Supplementary file6 (DOCX 13 kb)

## Data Availability

Data not here published are obtainable on reasonable request from the corresponding author.
