# Supplementary figures and images for: Effectiveness and safety of fluocinolone acetonide intravitreal implant in diabetic macular edema patients considered insufficiently responsive to available therapies (REACT): a prospective, non-randomized, and multicenter study
Source: Int Ophthalmol. 2023 Sep 12;43(12):4639–49. doi: 10.1007/s10792-023-02864-2 (PMC10724319; doi:10.1007/s10792-023-02864-2)

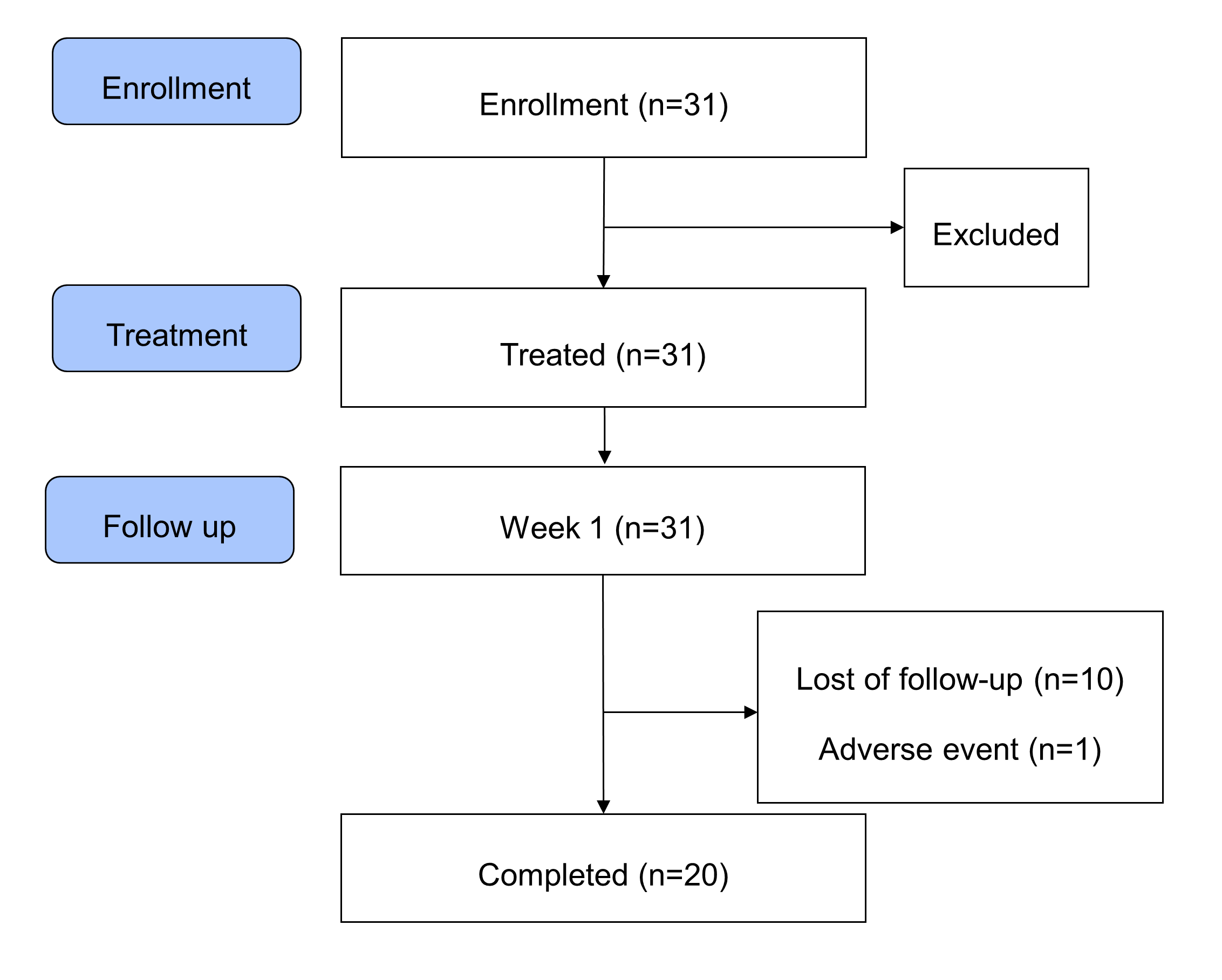

Supplement: Supplementary file 1 — Study Flowchart. 31 patients were included in the study from September 26, 2017, to October 30, 2018, and all patients were treated. The patients participated in this study between 26 September 2017 (first consent) and 16 February 2022 (last follow-up visit). Eleven did not complete the study: 10 loss of follow-up and 1 due to an adverse event. [file 10792_2023_2864_MOESM1_ESM.tif]

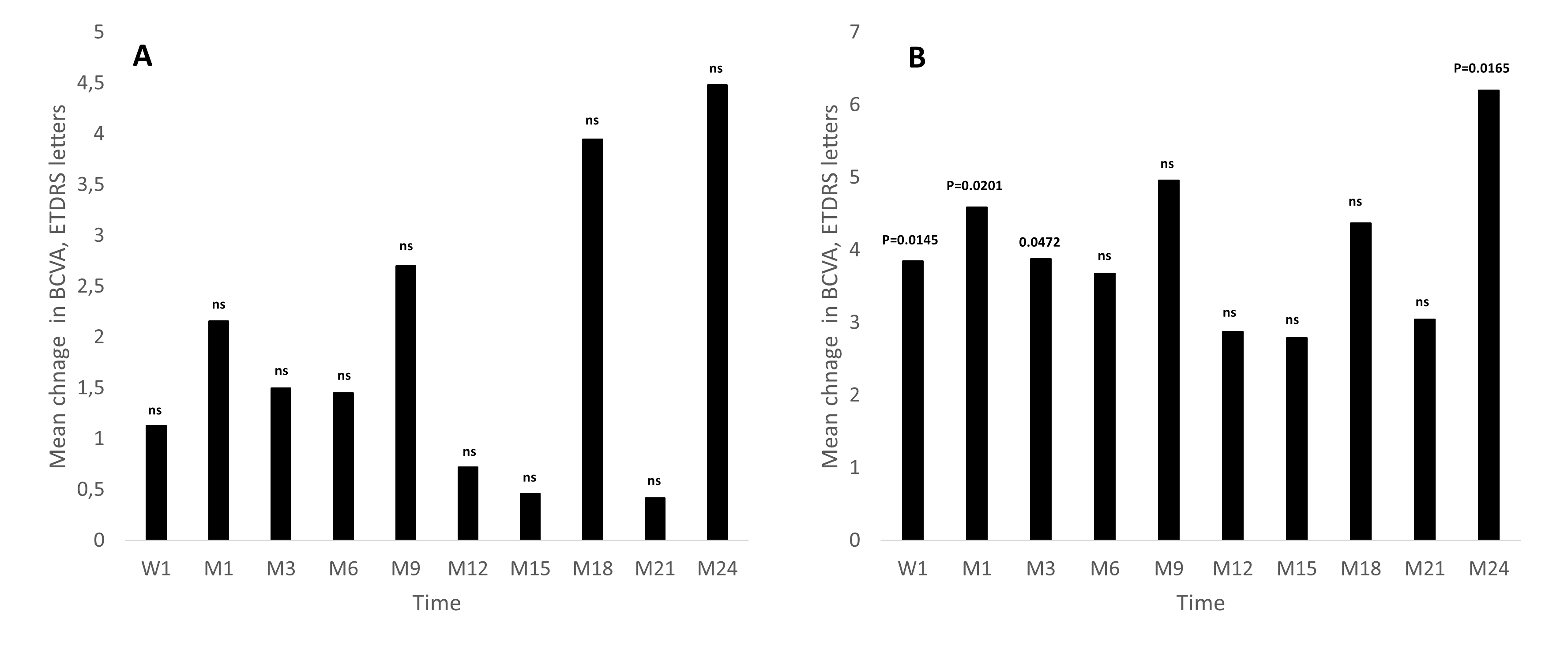

Supplement: Supplementary file 2 — Mean change in best corrected visual acuity (BCVA) throughout study follow-up in the overall study population (A) and in the eyes with a baseline BCVA < 70 ETDRS letters. Statistically significance was calculated by using paired sample two-tailed t test or Wilcoxon test, as appropriate. BCVA: Best corrected visual acuity; ETDRS: Early Treatment Diabetic Retinopathy Study; ns: Not significant; W: Week; M: Month. [file 10792_2023_2864_MOESM2_ESM.tif]

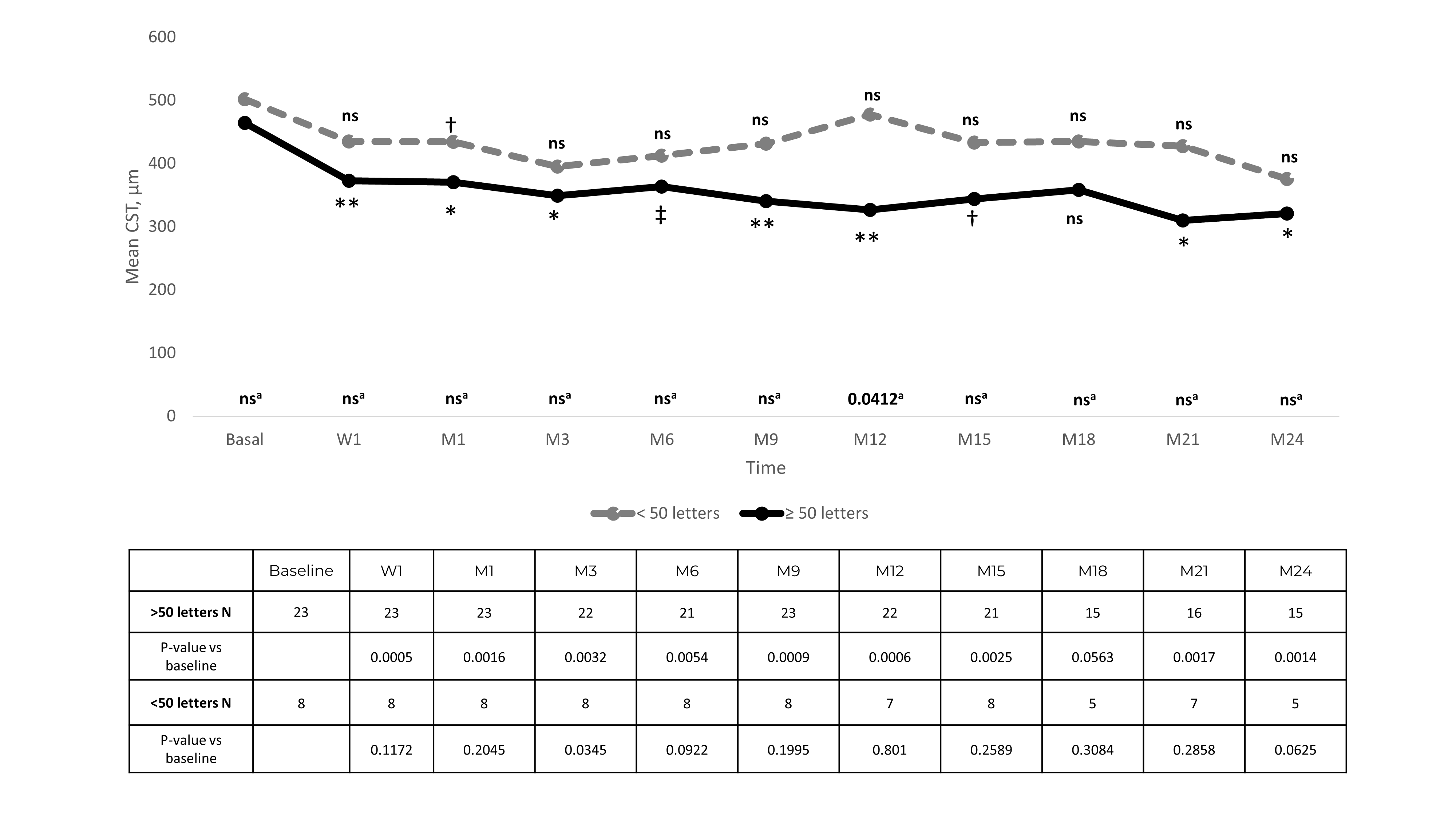

Supplement: Supplementary file 3 — A comparison of the mean central subfoveal thickness CST throughout study follow-up in the eyes with a baseline BCVA < 50 ETDRS letters (8 eyes; gray columns) and those with a baseline BCVA ≥50 ETDRS letters (23 eyes; black columns). Intragroup statistically significance was calculated by using the Wilcoxon test. Between group differences were calculated with the independent-samples t test. CST: Central subfoveal thickness; MV: Macular volume; W: Week; M: Month; ns: Not significant. a Between group significance, †p<0.05 from baseline. ‡p < 0.01 from baseline. *p < 0.005 from baseline. **p < 0.001 from baseline. [file 10792_2023_2864_MOESM3_ESM.tif]
