# Supplementary material for: Effectiveness and safety of fluocinolone acetonide intravitreal implant in diabetic macular edema patients considered insufficiently responsive to available therapies (REACT): a prospective, non-randomized, and multicenter study
Source: Int Ophthalmol. 2023 Sep 12;43(12):4639–49. doi: 10.1007/s10792-023-02864-2 (PMC10724319; doi:10.1007/s10792-023-02864-2)
Supplement: Supplementary file 4 — Supplementary file4 (DOCX 15 kb) [file 10792_2023_2864_MOESM4_ESM.docx]

Table S1. Main inclusion and exclusion criteria.

| Inclusion criteria | Exclusion criteria |
| --- | --- |
| Patients ≥18 years of age, of either sex that had signed informed consent.  DME based on investigator’s clinical evaluation and demonstrated using fundoscopic photography and SD-OCT.  Patients considered as insufficiently responsive as defined as having undergone other previous treatments, including at least 3 anti-VEGF injections in the last 6 months, and the following:   - Zeiss Cirrus (Meditec, Dublin, CA, USA) or ≥ 305μm in women and ≥ 320 μm in men in Heidelberg Spectralis (Heidelberg Engineering Inc, MA, USA), or equivalent accordingly with Tocon or Swept Source (Triton, Topcon Co, Topcon Corporation, Japan), in the study eye as measured using SD-OCT - Vision impairment (20/50 to 20/400 using Snellen visual acuity equivalent) related to DME whether in the Investigator’s opinion a further improvement was possible.   Willingness to comply with the investigators and protocol indications | IOP > 21 mmHg at screening in the study eye.  Historical rise in IOP > 25 mmHg following treatment with an intravitreal corticosteroid in the study eye.  Use of ≥ 2 active agents as IOP-lowering medications to control IOP at screening in the study eye.  Patients that had vitreomacular traction in DME and opaque media in the study eye.  Patients with severe proliferative diabetic retinopathy that required pan retinal photocoagulation in the study eye.  Patients diagnosed with active angiographic central macular ischemia prior to screening in the study eye.  Patients that received pan retinal photocoagulation or undergone cataract surgery in the 3 months prior to the screening visit in the study eye.  Patients with contraindications:   - Presence of pre-existing glaucoma. - Active or suspected ocular or periocular infection. - Hypersensitivity to the active agent or to one of the excipients   Pregnant or child-bearing potential women who did not want to use contraception methods during the study period |

DME: Diabetic macular edema; SD-OCT: Spectral domain optical coherence tomography; anti-VEGF: Vascular endothelial growth factor inhibitors; IOP: Intraocular pressure.
