# Supplementary material for: Effectiveness and safety of fluocinolone acetonide intravitreal implant in diabetic macular edema patients considered insufficiently responsive to available therapies (REACT): a prospective, non-randomized, and multicenter study
Source: Int Ophthalmol. 2023 Sep 12;43(12):4639–49. doi: 10.1007/s10792-023-02864-2 (PMC10724319; doi:10.1007/s10792-023-02864-2)
Supplement: Supplementary file 5 — Supplementary file5 (DOCX 17 kb) [file 10792_2023_2864_MOESM5_ESM.docx]

Table S2. A comparison of the mean change in best corrected visual acuity (BCVA) throughout study follow-up between the eyes with a baseline BCVA< 50 ETDRS letters and those with a baseline BCVA ≥50 ETDRS letters.

|  | Baseline BCVA* < 50 letters (n=8) | | | Baseline BCVA* ≥50 letters (n=23) | | | Difference between groups | |
| --- | --- | --- | --- | --- | --- | --- | --- | --- |
|  | n | Mean (95% CI) difference from baseline | p | n | Mean (95% CI) difference from baseline | p | Mean (95%CI) | p |
| Week 1 | 23 | 5.9 (-0.5 to 12.2) | 0.0657 | 8 | -0.52 (-6.7 to 5.6) | 0.4611 | -6. 3 (-17.2 to 4.5) | 0.2430 |
| Month 1 | 23 | 8.6 (-1.4 to 18.6) | 0.1250 | 8 | -0.09 (-6.0 to 5.8) | 0.4567 | -8.7 (-19.9 to 2.5) | 0.1225 |
| Month 3 | 22 | 5.4 (-2.8 to 13.6) | 0.1652 | 8 | 0.09 (-6.0 to 6.2) | 0.5901 | -5.3 (-16.2 to 5.6) | 0.3279 |
| Month 6 | 21 | 8.0 (-5.7 to 21.7) | 0.2098 | 8 | -1.1 (-7.0 to 4.9) | 0.6492 | -9.1 (-21.0 to 2.9) | 0.1304 |
| Month 9 | 22 | 16.0 (0.8 to 31.2) | 0.0417 | 8 | -2.3 (-8.3 to 3.7) | 0.4402 | -18.3 (-30.8 to -5.8) | 0.0057 |
| Month 12 | 22 | 16.9 (0.9 to 32.8) | 0.0417 | 7 | -4.4 (-12.6 to 3.8) | 0.2752 | -21.3 (-37.5 to -5.1) | 0.0121 |
| Month 15 | 21 | 19.4 (0.6 to 38.3) | 0.0451 | 7 | -5.9 (-14.8 to 3.0) | 0.1852 | -25.3 (-43.1 to -7.6) | 0.0070 |
| Month 18 | 16 | 12.6 (-11.4 to 36.6) | 0.2183 | 5 | 1.25 (-5.3 to 7.8) | 0.6920 | -11.4 (-23.5 to 3.8) | 0.1336 |
| Month 21 | 16 | 11.3 (-4.5 to 27.1) | 0.1313 | 7 | -4.1 (-13.6 to 5.5) | 0.3821 | -15.4 (-34.8 to 4.0) | 0.1139 |
| Month 24 | 15 | 9.8 (-14.1 to 33.7) | 0.3186 | 5 | 3.0 (-5.6 to 11.6) | 0.0857 | -6.8 (-25.3 to 11.7) | 0.4537 |

* Early Treatment Diabetic Retinopathy Study (ETDRS) letters.

CI: Confidence interval.
