# Supplementary material for: Effectiveness and safety of fluocinolone acetonide intravitreal implant in diabetic macular edema patients considered insufficiently responsive to available therapies (REACT): a prospective, non-randomized, and multicenter study
Source: Int Ophthalmol. 2023 Sep 12;43(12):4639–49. doi: 10.1007/s10792-023-02864-2 (PMC10724319; doi:10.1007/s10792-023-02864-2)
Supplement: Supplementary file 6 — Supplementary file6 (DOCX 13 kb) [file 10792_2023_2864_MOESM6_ESM.docx]

Table S3. Overview of the treatments for diabetic macular edema that started throughout the study.

|  | Total N=31  *m/**p (%) |
| --- | --- |
| **Intravitreal Injection in the study eye** | 114 / 19 (61.3%) |
| Number of intravitreal injections, mean (SD) | 6.0 (6.2) |
| Aflibercept | 48 / 14 (73.7%) |
| Ranibizumab | 51 / 9 (47.4%) |
| Dexamethasone | 13 / 9 (47.4%) |
| Triamcinolone | 1 / 1 (5.3%) |
| Fluocinolone acetonide | 1 / 1 (5.3%) |
| **Intravitreal Injection (Anti-VEGF treatment)** | 99 / 15 (78.9%) |
| Number of anti-VEGF treatment, mean (SD) | 6.6 (6.3) |
| Aflibercept | 48 / 14 (93.3%) |
| Ranibizumab | 51 / 9 (60.0%) |
| **Intravitreal Injection (corticoid)** | 15 / 11 (57.9%) |
| Number of intravitreal injections, mean (SD) | 1.4 (0.7) |
| Dexamethasone | 13 / 9 (81.8%) |
| Triamcinolone | 1 / 1 (9.1%) |
| Fluocinolone acetonide | 1 / 1 (9.1%) |

*m = number of medications by category

**p = number of patients by category, percent calculated as [(n / N) x100], where N is the total number of patients.

SD: Standard deviation; anti-VEGF: Vascular endothelial growth factor inhibitor.
